# Exploiting macro- and micro-structural brain changes for improved Parkinson’s disease classification from MRI data

**DOI:** 10.1038/s41531-024-00647-9

**Published:** 2024-02-26

**Authors:** Milton Camacho, Matthias Wilms, Hannes Almgren, Kimberly Amador, Richard Camicioli, Zahinoor Ismail, Oury Monchi, Nils D. Forkert, For the Alzheimer’s Disease Neuroimaging Initiative

**Affiliations:** 1 Biomedical Engineering Graduate Program, University of Calgary, Calgary, AB Canada; 2 Department of Radiology, University of Calgary, Calgary, AB Canada; 3 Alberta Children’s Hospital Research Institute, University of Calgary, Calgary, AB Canada; 4 Department of Pediatrics and Community Health Sciences, University of Calgary, Calgary, AB Canada; 5 Hotchkiss Brain Institute, University of Calgary, Calgary, AB Canada; 6 Department of Clinical Neurosciences, University of Calgary, Calgary, AB Canada; 7 Neuroscience and Mental Health Institute and Department of Medicine (Neurology), University of Alberta, Edmonton, AB Canada; 8 Department of Psychiatry, University of Calgary, Calgary, AB Canada; 9 College of Medicine and Health, University of Exeter, Exeter, UK; 10 Department of Radiology, Radio-oncology and Nuclear Medicine, Université de Montréal, Montréal, QC Canada; 11 Centre de Recherche, Institut Universitaire de Gériatrie de Montréal, Montréal, QC Canada

**Keywords:** Parkinson's disease, Diagnostic markers, Brain, Diagnostic markers, Parkinson's disease

## Abstract

Parkinson’s disease (PD) is the second most common neurodegenerative disease. Accurate PD diagnosis is crucial for effective treatment and prognosis but can be challenging, especially at early disease stages. This study aimed to develop and evaluate an explainable deep learning model for PD classification from multimodal neuroimaging data. The model was trained using one of the largest collections of T1-weighted and diffusion-tensor magnetic resonance imaging (MRI) datasets. A total of 1264 datasets from eight different studies were collected, including 611 PD patients and 653 healthy controls (HC). These datasets were pre-processed and non-linearly registered to the MNI PD25 atlas. Six imaging maps describing the macro- and micro-structural integrity of brain tissues complemented with age and sex parameters were used to train a convolutional neural network (CNN) to classify PD/HC subjects. Explainability of the model’s decision-making was achieved using SmoothGrad saliency maps, highlighting important brain regions. The CNN was trained using a 75%/10%/15% train/validation/test split stratified by diagnosis, sex, age, and study, achieving a ROC-AUC of 0.89, accuracy of 80.8%, specificity of 82.4%, and sensitivity of 79.1% on the test set. Saliency maps revealed that diffusion tensor imaging data, especially fractional anisotropy, was more important for the classification than T1-weighted data, highlighting subcortical regions such as the brainstem, thalamus, amygdala, hippocampus, and cortical areas. The proposed model, trained on a large multimodal MRI database, can classify PD patients and HC subjects with high accuracy and clinically reasonable explanations, suggesting that micro-structural brain changes play an essential role in the disease course.

## Introduction

Parkinson’s disease (PD) is a severe and heterogeneous progressive disease recognized as the second-most common neurodegenerative disorder. It affects 2–3% of the population aged 65 years or older^1^ with an estimate of seven to ten million people worldwide diagnosed with the disease^2^. PD is pathophysiologically characterized by the loss of dopaminergic neurons in the substantia nigra and the accumulation of alpha-synuclein aggregates within cells^3^. As the disease progresses, this accumulation can spread to wider regions of the cortex^4^. Despite the improving understanding of the disease, the exact causes of PD are still not well understood. Currently, PD is mainly diagnosed following clinical guidelines that involve assessing the presence of bradykinesia (slowness of movement) as well as at least one of the other primary motor symptoms such as tremor, rigidity, and postural instability^5^. Additional motor and non-motor symptoms also contribute to the overall clinical picture^6^. However, diagnostic accuracy based on clinical guidelines can vary from 73.8 to 83.9%, depending on the neurologist’s experience^7,8^. Therefore, ongoing research is aiming to identify accurate and reproducible biomarkers for PD diagnosis. Neuroimaging techniques, such as magnetic resonance imaging (MRI) and positron emission tomography, have been studied extensively for this purpose due to their ability to capture the structure and function of the brain with high sensitivity and resolution. For example, T1-weighted MRI has shown potential capturing macro-structural changes in the brain^9–14^. In the context of micro-structural changes, diffusion-tensor MRI (DTI) allows for in-vivo characterization of diffusivity in brain tissues through its sensitivity to the Brownian motion of water molecules. Water tends to diffuse mainly along the axons so that DTI can be employed to visualize white matter tracts throughout the brain^15^. This property makes it particularly advantageous for studying white matter integrity in different neurological diseases^16^, but may also be used to uncover potential micro-structural changes of gray matter structures in neurodegenerative diseases like PD^17^. The main DTI parameters are mean diffusivity (MD), which measures the degree of tissue water diffusivity, fractional anisotropy (FA), axial diffusivity (AD), and radial diffusivity (RD), which are related to the axonal and myelin structure^18–20^. Nevertheless, visual identification of such macro- and micro-structural changes associated with PD, especially in the early disease phase, can be challenging and time-consuming, even for the most experienced neuroradiologist^7^.

Supervised machine learning approaches seek to automatically detect relevant patterns within a high-dimensional feature space. They do this by learning from the training data provided and creating an association with the ground truth labels provided as a reference. These patterns can then be utilized to classify new, previously unseen cases^21^. Machine learning models have been created for many clinical tasks by leveraging high-dimensional data for individual-level classification^22^. Following this principle, machine learning models have also been developed for PD classification making use of macro-structural neuroimaging data with accuracy levels above 70%^13,14,23–31^. Similarly, micro-structural information extracted from DTI has also been explored for this purpose (77.44–97.7% accuracy)^13,30–37^. So far, only two previous studies investigated the benefit of using both, micro- and macro-structural MRI, for PD detection with machine learning^13,30^, achieving accuracies of 92.3 and 95.1%. Most of the previous studies for both uni- and multimodal MRI only used small sample sizes and followed a traditional machine learning approach, which typically includes image preparation through hand-crafted preprocessing techniques, feature extraction, and training and testing a classical machine learning model like a support vector machine and elastic-net regression, which are typically evaluated using a cross-validation strategy. A significant drawback of these techniques is that the features must be explicitly designed before training, which could result in missing crucial features. Convolutional neural networks (CNN), a special type of deep learning network, overcome this problem by automatically learning a representation of the input data with multiple levels of abstraction through an iterative training process. However, CNNs typically need large amounts of data to optimize these models without overfitting, which may be one of the reasons that there are no CNN models for PD classification making use of multimodal data to date.

In summary, previous uni- and multimodal studies described have been only tested and evaluated using a rather small number of PD patients and healthy participants ranging from 40 to 374 subjects, using data that is imbalanced and collected within a single study. Additionally, most previous machine learning models failed to provide explanations for their model’s predictions, which is crucial for building trust in computer-aided diagnosis systems and also to aid biomarker discovery.

To overcome these limitations, we collected the largest balanced multimodal MRI dataset containing both T1-weighted and DTI scans from PD patients and matched healthy controls from multiple studies, resulting in a total of nearly 1300 subjects included. Briefly described, for morphological analysis of the brain tissue, the T1-weighted MRI data were affinely and non-linearly registered to an atlas. The non-linear transformation was consequently used to compute the corresponding log-Jacobian maps, which encode volumetric differences between the atlas and a subject’s brain at a voxel level. Parametric maps of MD, FA, AD, and RD were computed based on the DTI datasets and also non-linearly registered to an atlas. The resulting six imaging maps, namely the affinely registered T1-weighted images (Aff), the log-Jacobian maps (Jac), and the six DTI parametric maps (MD, FA, AD, and RD) together with age and sex as parameters were used to train and evaluate a 3D CNN model (**CNN**_**COMBINED**_) for PD classification. For comparison purposes, six additional CNN models were trained using the single imaging maps only (**CNN**_**AFF**_**, CNN**_**JAC**_**, CNN**_**MD**_**, CNN**_**FA**_**, CNN**_**AD**_**, CNN**_**RD**_), one additional CNN model using all DTI parametric maps but none of the T1-derived imaging maps (**CNN**_**DTI**_), as well as one classical random forest machine learning model using image-derived phenotypes from the T1-weighted and DTI data (**RF**_**COMBINED**_). All machine learning models were trained using the same 75%/10%/15% train/validation/test split stratified by diagnosis, sex, age, and study. Saliency maps were used to explain the predictions by highlighting the regions that were most influential for the classification of an individual patient.

## Results

### Evaluation of models

The prediction metrics in terms of area under the receiver operating characteristic curve (ROC-AUC), specificity, and sensitivity for all trained models are provided in Table 1. The model using the combination of all input imaging maps (**CNN**_**COMBINED**_) achieved the overall best results (0.89 ROC-AUC, 80.8% accuracy, 82.4% specificity, and 79.1% sensitivity) with respect to the ROC-AUC metric. Therefore, this model was chosen to generate the saliency maps for further post-hoc analysis using the cortical and subcortical Harvard-Oxford atlas brain regions^38–41^ (HO). Overall, the **CNN**_**COMBINED**_ model performed significantly better than the models using only the information from the T1-weighted MRI data (**CNN**_**JAC and**_ **CNN**_**AFF**_, *p* < 0.05). However, it did not perform significantly better than the individual DTI (**CNN**_**FA, MD, RD, and AD**_) and random forest (RF_COMBINED_) models.

**Table 1:** Table of results

Model	ROC-AUC	ACC	SPE	SEN
CNN_COMBINED_	**0.89**	80.8%	82.4%	79.1%
CNN_DTI_	0.86	78.1%	79.3%	76.9%
CNN_FA_	0.86	81.3%	78.3%	82.6%
CNN_MD_	0.85	77.1%	79.3%	74.7%
CNN_RD_	0.84	77.6%	77.3%	78.0%
CNN_AD_	0.85	77.6%	79.3%	75.8%
CNN_JAC_^a^_(P 0.032)_	0.83	73.9%	67.0%	81.3%
CNN_AFF_^a^_(P 0.035)_	0.85	76.0%	85.5%	65.9%
RF_COMBINED_ (159 FEATURES)	0.85	79.7%	76.2%	79.8%

*ROC-AUC* area under the receiver operating characteristic curve, *ACC* accuracy, *SPE* specificity, *SEN* sensitivity.

^a^Significant difference (*P* < 0.05) with CNN_COMBINED_.

### Explainability analysis

**Fig. 1:**
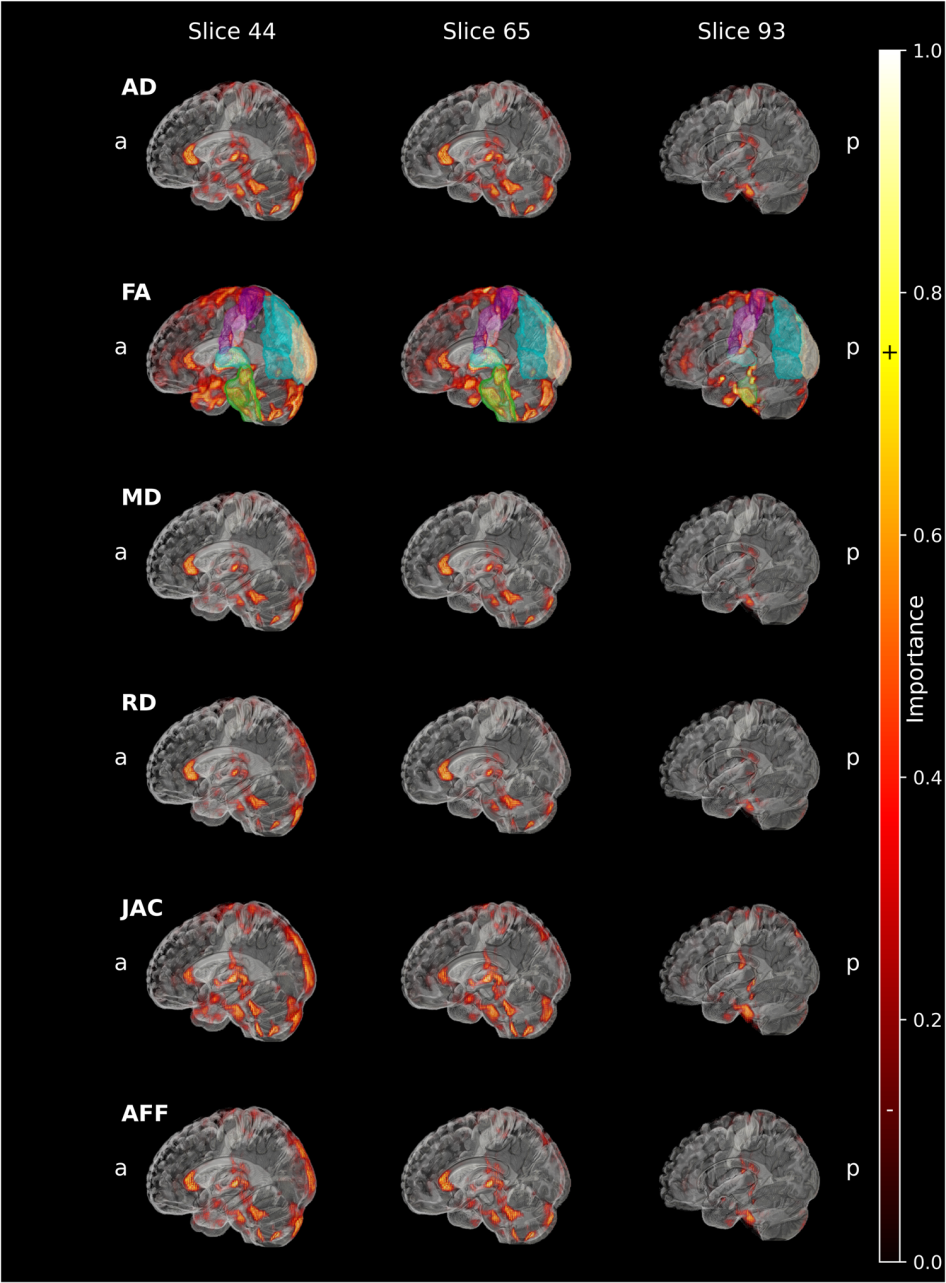
Average saliency maps of the correctly classified PD patients overlayed on the PD25 atlas for the independent input maps. AD axial diffusivity, FA fractional anisotropy, MD mean diffusivity, RD radial diffusivity, JAC Jacobian, AFF affine, a anterior, p posterior. To help link anatomical regions shown in Fig. 2 to the most important saliency map regions shown here, a color coding was inserted in the FA slices. Cortical regions like the postcentral cortex are displayed in purple, the supramarginal gyrus and posterior division in pink, the lateral occipital cortex, superior and inferior divisions in teal, and the occipital pole in beige. Subcortical regions like the left and right thalamus are shown in turquoise and the brainstem in green.

Figure 1 shows the saliency maps derived from **CNN**_**COMBINED**_ classifications for the correctly diagnosed PD patients in the test set. The highlighted regions across the multiple input maps represent the regions used by the model to produce a classification, whereas a higher saliency value represents higher relevance for the model.

**Fig. 2:**
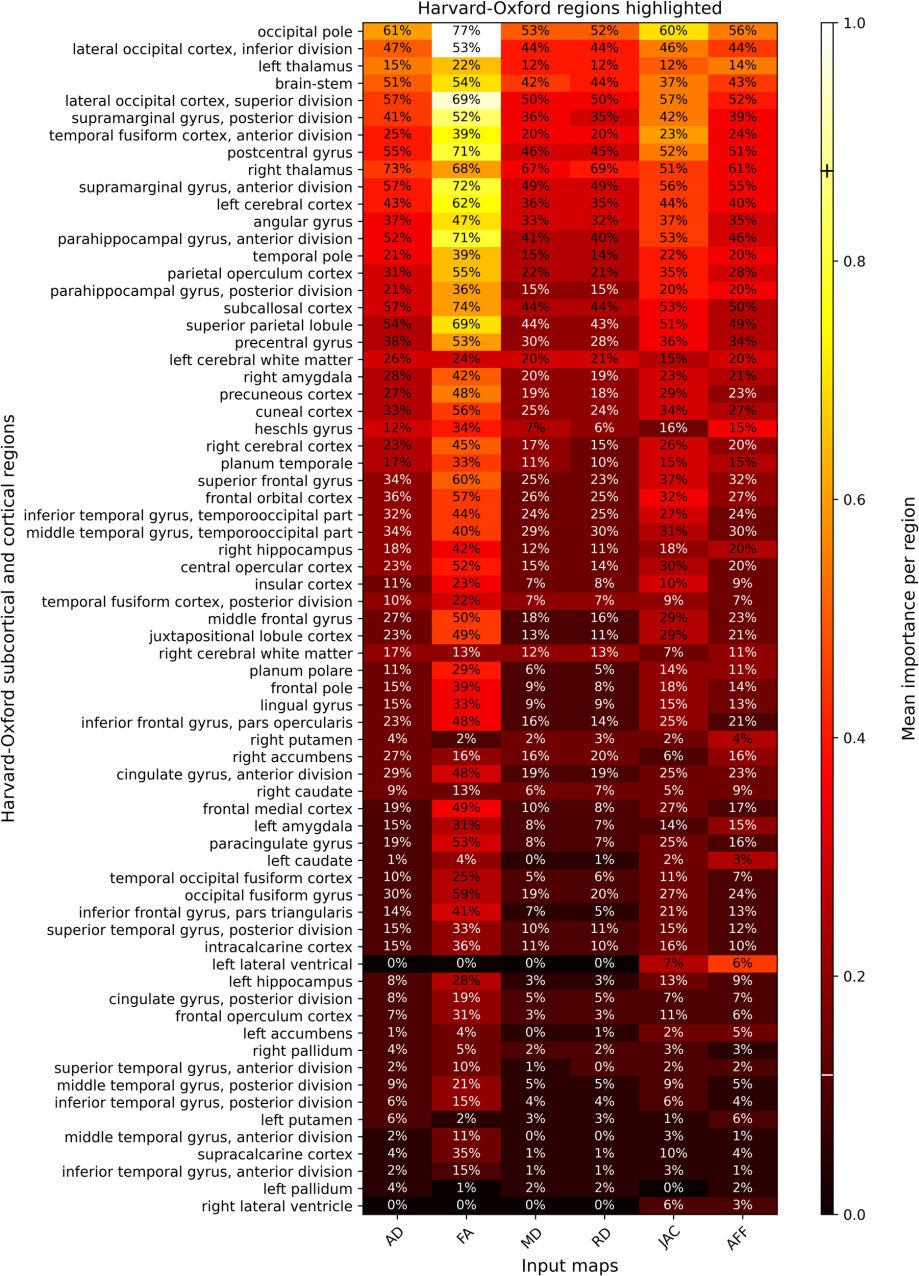
Heatmap of importance for each region of the Harvard-Oxford atlases. Intensity mean values are represented according to the color in each rectangle. Additionally, each region has a percentage of volume covered by the thresholded average saliency map of the correctly classified PD patients. Regions were ranked according to an average of the importance in each input map.

Figure 2 shows a heatmap of the average intensity and volume covered by the saliency maps for each atlas region across the multiple input images of the sub-cortical and cortical Harvard-Oxford brain atlases. Overall, FA was found to be the most salient image feature, followed by log-Jacobian morphological image feature. The brain regions identified as most important by the **CNN**_**COMBINED**_ model included subcortical regions like the brainstem, bilateral thalamus, right amygdala, right hippocampus, and cortical regions including the occipital cortex, supramarginal gyrus, postcentral and precentral gyrus, temporal cortex, parahippocampal gyrus, angular gyrus, parietal operculum cortex, superior parietal lobe, and subcallosal cortex. For the random forest machine learning model, the most important features selected by the RELIEFF feature selection method are generally in line with the results of the saliency maps computed from the **CNN**_**COMBINED**_ and are shown in Supplementary Table 1.

**Fig. 3:**
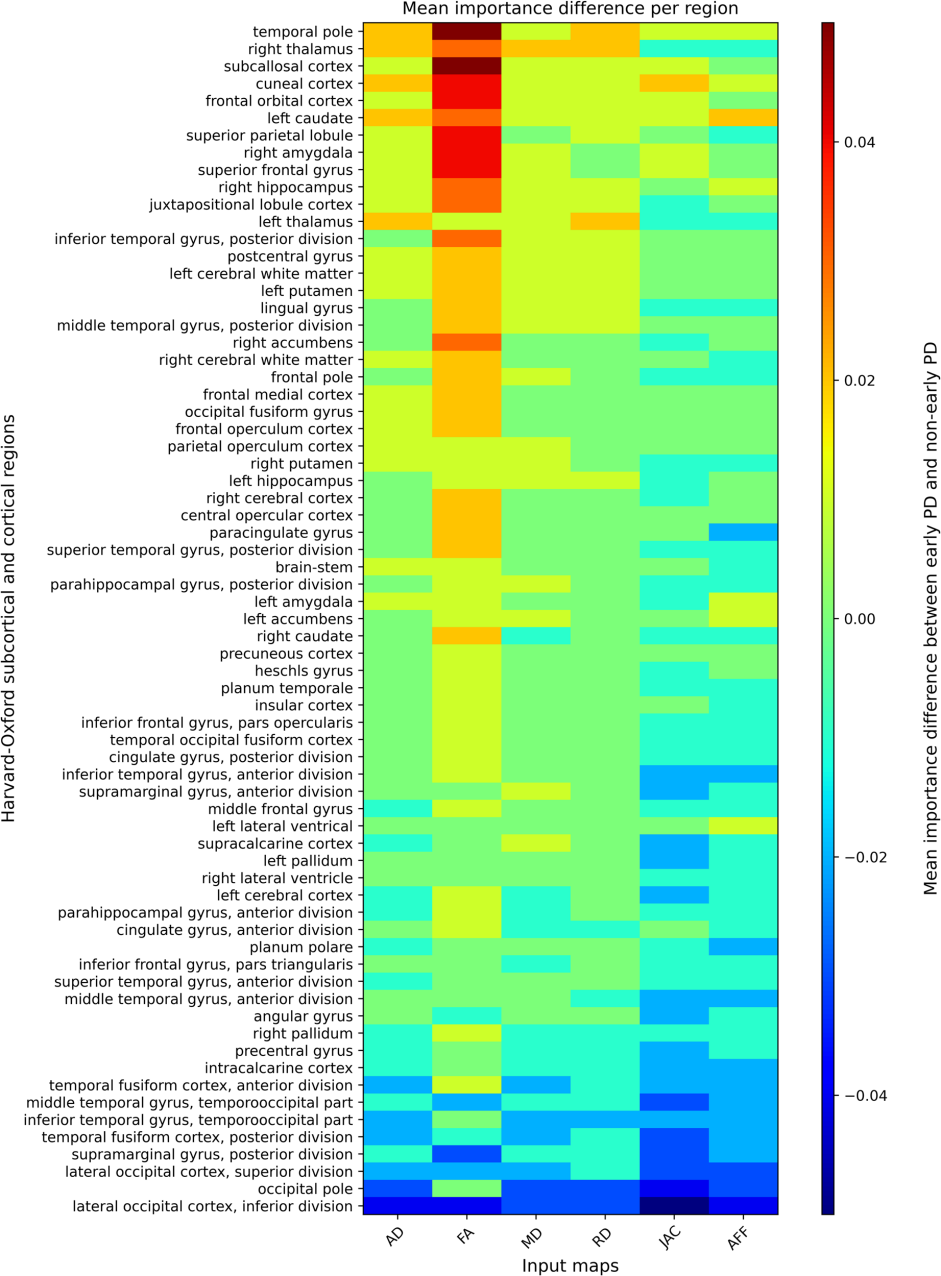
Heatmap of mean intensity importance difference for each region of the Harvard–Oxford atlases. Every color represents the difference between early and non-early subjects’ activations (e.g., right thalamus of early subjects minus right thalamus of non-early subjects) as indicated by the corresponding color bar.

Figure 3 shows the differences in mean intensity values when comparing early PD patients (Parkinson’s Progression Markers Initiative [PPMI] patients in the test set) and non-early PD patients (rest of the test set). Here, it is possible to identify a pattern with early PD patient’s saliency maps having slightly higher activations in the DTI maps when compared to the PD patients in later disease stages. The opposite can be observed for the later disease stage patients’ activations, which are slightly higher for morphological features derived from the T1-weighted scans when compared to the activations of early patients.

## Discussion

The main contribution of this study is the development of an accurate and explainable deep learning model for the image-guided diagnosis support of Parkinson’s disease based on high-resolution, multimodal MRI data. This model was trained and tested using one of the biggest multi-study databases comprising macro-structural (T1-weighted MRI) and micro-structural (DTI) information from PD patients and HC subjects. Saliency map explanations were then computed to identify the most important regions from each input map for the decision task.

In the present study, the proposed multimodal deep learning model (**CNN**_**COMBINED**_) performed better than all uni-modal CNN models and the classical machine learning model, although not reaching significance in all cases. Deep learning outperforming traditional machine learning approaches is expected and in line with the current literature^13,23,24,26–30,33–35,37^. The improved performance may be partly explained by the ability of CNN models to automatically extract the important features from the complete set of 3D input maps, and thus learning from every voxel in the image. In contrast, manual feature engineering may cause important information to get missed in the process. Although it is possible that more sophisticated feature engineering techniques could have benefitted the performance of the classical machine learning model, it is unlikely that adding more and other brain regions would have boosted the comparison method beyond the accuracy of the deep learning method. The significantly better results of the **CNN**_**COMBINED**_ model compared to the models trained using information from the T1-weighted data only are likely directly due to the inclusion of DTI maps, which were found to be more important for the task of PD prediction in the combined model. However, it is also possible that some participants are easier to differentiate with information from certain input maps but not from others. This could also explain how the model including all available maps achieved higher performance than the models trained with the best individual map FA maps (i.e., **CNN**_**FA**_), highlighting the benefits of training a model that combines macro- and micro-structural imaging. Overall, the models trained using micro-structural information performed better than the models trained using macro-structural information only. This implies that the models can identify important micro-structural differences, which are not present in macro-structural data. Nevertheless, considering that T1-weighted images are part of virtually any MRI protocol, it appears reasonable to still include them as inputs of the model, even if the enhancements they offer are modest at a group level. Therefore, this study investigates the use of a combination of micro- and macro-structural information as a multi-channel input to train a CNN for PD classification.

In the context of deep learning, a simple and accurate model is also typically assumed to be more generalizable and easier to explain, which is particularly important for a multimodal approach where data complexity is increased. The proposed deep learning model is relatively lightweight, containing only 2,982,369 trainable parameters. In contrast, larger CNN models or ensembled approaches ranging from 16 to 89 million trainable parameters (not to mention trained with far smaller sample sizes) as previously described in literature^24,27,35,37^ could lead to less interpretable results and are more vulnerable to overfitting.

The prediction performance of the proposed model on unseen data is in the range of previously reported accuracies based on macro-structural brain data, micro-structural brain imaging, and a combination of both (ranging from 71.5% to 100%; accuracy reported due to the lack of AUC in all works)^13,23,24,26–30,33–35,37,42^. While accuracy levels close to 100% in previous works seem impressive, some of these studies have not been peer-reviewed, so that the results described should be taken with a grain of salt, while in other cases, such high accuracies were achieved on very small and imbalanced datasets. This result inflation is a well-known challenge in neuroimaging research, which should be considered^43,44^. In contrast, the machine learning models created for this study were trained on a much richer and balanced database of Parkinson’s disease patients and healthy control subjects using well-established and common pipelines for image registration and DTI map calculation. The results achieved on the diverse test set (which also contained data from the eight different studies) with robust processing pipelines suggest that our model generalizes well to unseen data. Moreover, the complexity of the datasets used for this study resembles that of a current real clinical scenario where it is essentially impossible to collect highly homogeneous data. This data diversity can enhance the generalizability of our classifier when applied in various clinical contexts. Nevertheless, to address potential biases resulting from dataset imbalances between studies, we applied weights to the loss function during training. Thus, our model stands out as an important step towards a computer-aided diagnosis tool for PD because of its ability to identify micro- and macro-structural differences between the groups despite the heterogeneity of the data.

This study applies methods from the explainable artificial intelligence domain to complex CNN models for PD classification from multimodal data. CNN models are often considered “black box” models, which suggests that it is challenging to understand how they arrive at their predictions. Through explainable techniques, we can overcome the currently existing clinical adoption barrier in precision medicine. In this context, the developed model could be employed by medical professionals to evaluate the validity of the models’ predictions and use the generated saliency maps for multimodal biomarker identification.

In general, the salient brain areas found in this study are consistent with our existing understanding of Parkinson’s disease and how it affects the brain, both at a micro- and macro-structural level. More in detail, wide-spread morphological changes were identified in subcortical and cortical regions as important by our proposed combined model (**CNN**_**COMBINED**_). The model also gave high importance to the FA DTI maps. This finding is in line with multiple previous studies that identified significant brain changes associated with Parkinson’s disease in FA maps^36,37,45,46^. Jacobian maps were given the second highest importance, despite not leading to such high classification accuracies when used without the DTI data, emphasizing the importance of a multimodal classification approach. The associated subcortical regions included the brainstem, thalamus, amygdala, and hippocampus. The brainstem is considered a vulnerable structure, which is known to be affected very early in neurodegenerative diseases like PD^47^. The thalamus is known to suffer a 40–50% neuronal loss in PD patients^48^, which could explain its appearance in the saliency maps. Nevertheless, how this contributes to the pathology is not yet clearly understood^49^. Changes in the amygdala and hippocampus have also been reported in the literature^50–54^ and have been linked to non-motor symptoms of Parkinson’s disease, which can present early in the disease. The occipital cortex (occipital pole, lateral inferior and superior divisions, and cuneus cortex), temporal cortex (temporal pole, temporal fusiform cortex, and parahippocampal gyrus anterior and posterior divisions), parietal cortex (supramarginal anterior and posterior gyrus, postcentral and precentral gyrus, angular gyrus, superior lobule, operculum and precuneus cortex), and subcallosal cortex were identified as the most important cortical regions by our model. These findings are also in line with current literature. For example, micro- and macro-structural differences of the temporal cortices in PD compared with HC have been reported^55^. Moreover, macro-structural differences of the occipital and parietal cortices have been found in previous studies^56^. Additionally, micro-structural changes of the subcallosal cortex have been linked to non-motor symptoms in PD^57^. While the brain atlas regions used to analyze the saliency maps include two labels for the white matter in each hemisphere, aiding in the identification of important regions. However, these relatively large regions may not be specific enough for detailed post-hoc analysis of white matter tracts, an aspect that could be more thoroughly explored in future research. Nevertheless, due to the voxel-wise nature of the CNN model, it is able to use detailed data from white matter regions. More generally, the brain areas that our model identified as important are consistent with Braak’s staging model^4^, which proposes that alphasynuclein deposition moves in ascending manner, starting in the brainstem and moving through the subcortex (i.e., subcortical regions) and mesocortex (parahippocampal gyrus) to ultimately the neocortex (making up to 90% of the human cortex).

**Fig. 4:**
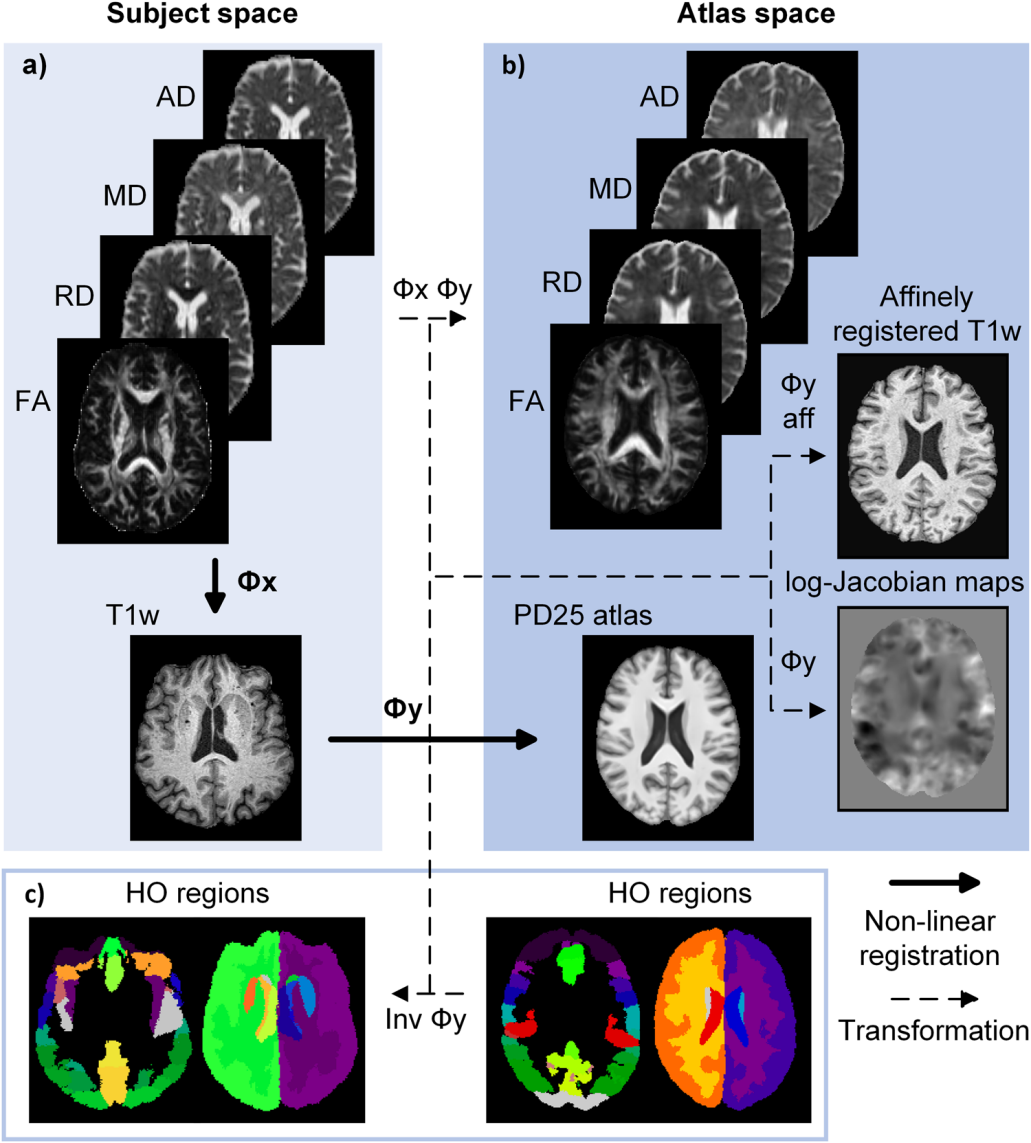
Preprocessing pipeline. **a** Illustrates part one of the preprocessing with the computation of the DTI metrics and the registration to the T1-weighted (T1w) MRI subject space. **b** Depicts the registration of the subject’s T1-weighted MRI to the PD25 atlas; the transformation of the DTI metrics to the atlas space, and the computation of the affinely registered T1-weighted MRI and the log-Jacobian maps. **c** Showcases the use of the inverse *Φ*y transformation to warp the atlas anatomical regions to the individual’s T1-weighted MRI space.

Model explanations were independently computed for early (i.e., PPMI study) and non-early patients in the test set to investigate how each imaging modality affected their predictions. We found a general increase in importance of the DTI maps, in particular FA, and a decrease of importance for the morphological T1-weighted image information for the early patients from the test set as depicted in Fig. 4. This is in line with the current clinical understanding that micro-structural changes precede macro-structural alterations in neurodegenerative diseases^58,59^. The early participants were selected from PPMI, which is a multicenter study collected without a harmonized imaging protocol. Given the large number of centers contributing data to PPMI, it is unlikely that the differences found are simply due to site differences or biases.

Many of the brain regions identified by the CNN model were also found to be important by the RELIEFF feature selection technique (*e.g*., brainstem, thalamus, amygdala, subcallosal cortex, postcentral and precentral gyrus, as well as multiple regions from the temporal and occipital lobes). Nonetheless, the reduced performance when compared to the **CNN**_**COMBINED**_, demonstrates how CNN models can extract more meaningful micro- and macro-structural changes than those extracted through feature engineering.

Some limitations of this work need to be mentioned. First, although using data from multiple centers generally improves the reliability of the findings and the generalizability of the model, the retrospective nature of the data collection also results in differences in the specific inclusion/exclusion criteria, psychiatric co-morbidities, cognitive impairment (including dementia), and overall PD diagnosis criteria. We mitigated these effects by including approximately the same proportion of subjects with respect to group, age, and sex from each of the included studies whenever possible as part of the training, validation, and testing splits. Furthermore, a weight was given to each subject during training to help mitigate negative effects of an imbalanced dataset by putting more weight on individual samples from underrepresented studies/groups (i.e., the PD patients from the study with less PD subjects would have a higher weight and vice versa). Nevertheless, additional thorough prospective research is needed to validate the proposed model placing special emphasis on the training weights and their effect on model performance. Additionally, while we rigorously and visually inspected all data for motion artifacts and excluded subjects with visible corruptions, we cannot fully rule out that more subtle motion artifacts survived our quality control. Due to the retrospective nature of this study, it was not possible to correct for motion prospectively or quantify any head motion during image acquisition so that we were limited to the manual quality control. Thus, the classifier developed may also make use of small motion artefacts that could affect the PD cohort more because of the tremor. It should also be mentioned that no advanced data harmonization approaches were utilized to remove potential site- or scanner-specific image data biases, which may still affect the otherwise quantitative DTI maps and log Jacobian maps to some extent. It is also important to highlight that the use of a combined multimodal approach can lead to scalability challenges since more time is required to preprocess and quality control the data for each imaging modality available to every individual subject. However, the pipeline used in this study is well-established and was found to be robust. Additionally, higher data dimensionality inherently results in more complex models, which can be harder to train and optimize, requiring additional computational resources. Thus, we selected a lightweight CNN, which was able to identify clinically reasonable important features and be explainable without compromising performance.

In conclusion, the present study demonstrates that multimodal MRI can be used and is beneficial to develop an explainable computer-aided diagnosis model based on a lightweight 3D CNN for Parkinson’s disease prediction. This model was trained using pre-processed, paired micro- and macro-structural MRI data from eight separate imaging studies, resembling real clinical scenario data. The model achieved performance in the range of literature values while providing clinically sound explanations for its predictions. The proposed model is not meant to replace but rather complement the decision-making process at the discretion and interpretation of experienced physicians^22^.

## Methods

### Datasets and subjects

Data were obtained from eight different studies comprising 632 PD patients and 668 control subjects (HC) (1300 participants in total). The studies included were the Parkinson’s Progression Markers Initiative (PPMI [www.ppmi-info.org]), the Canadian Consortium on Neurodegeneration in Aging (CCNA) COMPASS-ND^60^, PD-MCI Calgary^61^, PD-MCI Montreal^62^ (Longitudinal Study on Mild Cognitive Impairment in Parkinson’s Disease), Montreal Neurological Institute’s Open Science Clinical Biological Imaging and Genetic Repository (C-BIG [https://www.mcgill.ca/neuro/open-science/c-big-repository]), Hamburg dataset^63^, United Kingdom Biobank (UK Biobank [https://pan.ukbb.broadinstitute.org]), and healthy subjects only from the Alzheimer’s Disease Neuroimaging Initiative (ADNI [adni.loni.usc.edu]). For this secondary data use study, we included all participants that met the following criteria: (1) diagnosis of either PD or HC according to each individual study, (2) availability of both T1-weighted and DTI datasets from a 3T scanner (earliest available in case of longitudinal studies like PPMI, and only HC from ADNI with an age range of 65 ± 10 to balance the dataset), and (3) both preprocessed scans (T1-weighted and DTI scans) passing our quality control checks (See Preprocessing Section). The final database after preprocessing and quality controls included 1264 subjects (611 PD and 653 HC). Table 2 contains demographics, clinical, and diagnostic criteria information of the participants used for the present study. Supplementary Table 2 contains information regarding the inclusion and exclusion criteria of the different studies. All individual studies obtained ethical approval from their local ethics committee and ensured that all participants provided written informed consent following the guidelines set forth in the declaration of Helsinki. Moreover, the current study received approval from the Conjoint Health Research Ethics Board at the University of Calgary.

**Table 2:** Demographics of subjects included in the study

Study	Total Subjects (female %)		Age in Years Mean (std)		Duration of Disease in Years Mean (std)	H&Y Median		MoCA Mean (std)		MDS-UPDRS3 for PD (std)	Clinical Diagnostic Criteria
	PD	HC	PD	HC	PD	PD	HC	PD	HC		
PPMI	222 (34.6%)	64 (36.3%)	62.4 (9.4)	60.2 (10.8)	0.55 (0.56)	2	- -	27.37 (2.21)	28.22 (1.14)	20.96 (9.32)	MDS
COMPASS-ND	65 (32.3%)	-	69.1 (7.3)	-	-	2	-	24.23 (4.83)	-	9.07 (5.33)	MDS
PD-MCI Calgary	108 (34.2%)	42 (52.3%)	70.5 (6.2)	69.5 (6.7)	5.94 (4.51)	-	-	25.57 (2.94)	27.35 (1.94)	19.50 (12.54)	MDS
C-Big	82 (42.6%)	11 (81.8%)	64.1 (8.6)	64.2 (12.8)	-	-	-	-	-	38.88 (14.18)	MDS
Hamburg	49 (28.5%)	39 (37.5%)	65.4 (8.2)	62.5 (11.6)	12.82 (6.94)	2	-	-	-	27.10 (10.40)	UKBB
UK Biobank	42 (45.2%)	192 (40.1%)	69.4 (5.9)	(66.3) (7.6)	7.46 (6.62)	-	-	-	-	-	UKBB
PD-MCI Montreal	43 (39.5%)	20 (50.0%)	62.1 (5.7)	62.4 (6.2)	4.98 (3.80)	-	-	27.23 (1.99)	27.80 (1.93)	29.69 (8.20)	UKBB
ADNI	-	285 (65.6%)	-	67.7 (4.3)	-	-	-	-	-	-	-
Total	611 (37.6%)	653 (37.5%)	65.5 (8.7)	66.1 (7.6)	-	-	-	-	-	-	MDS and UKBB

*H&Y* Hoehn and Yahr rating scale, *MoCA* Montreal Cognitive Assessment test, *MDS* Movement Disorder Society clinical diagnosis criteria, *UKBB* United Kingdom Parkinson’s Disease Society Brain Bank clinical diagnostic criteria.

### MRI acquisition

MRI images were acquired using a variety of scanners and sequence parameters across the eight studies. All images were acquired from a range of MRI scanner manufacturers but all using a 3T magnetic field (Siemens [for seven studies], GE [for four studies], and Phillips [for two studies]). The T1-weighted images had varying slice and in-slice resolution, ranging from 0.94 to 2.0 mm, and most of the images were obtained in the sagittal plane. DTI scans had a slice thickness and in-slice resolution ranging between 2–5 and 1.875–2 mm, respectively; *b* values ranged from 0 to 2000; and number of directions of acquisition ranged from 15 to 114. More information about the imaging protocols used in each study is provided in Supplementary Table 3 in the Supplementary material.

### Preprocessing

Stringent quality control checks were performed on the raw data prior to preprocessing, these were performed through visual inspection to exclude MRI scans affected by movement, aliasing, ghosting, and other effects.

The three steps of the data pre-processing are summarized in Fig. 4. First, the TractoFlow pipeline^64^ was used for processing of the DTI data, including skull stripping, denoising, Eddy current correction, N4 bias correction, resampling to an isotropic resolution of 1 mm with linear interpolation, and computation of the four DTI metrics (i.e., FA, MD, RD, and AD). Similarly, individual T1-weighted images were brain extracted, denoised, N4 bias corrected, and resampled to have 1 mm isotropic voxels. Additionally, the extracted individual DTI metrics (using the B0 and FA together as the moving images) were non-linearly registered (*Φ*_x_ in Fig. 4) to the preprocessed T1-weighted MRI image (fixed image) of the same individual using ANTs^65^ (Fig. 4a). Briefly explained, this process included aligning the DTI datasets to the patient’s preprocessed T1-weighted scan using an affine transformation in the first step. This transformation was then used to initialize a non-linear registration^65^ as a second step. At this point, all transformed images were visually checked to identify any misaligned datasets. Next, the preprocessed T1-weighted image (moving image) was non-linearly registered (*Φ*_y_ in Fig. 4) to the Montreal Neurological Institute (MNI) PD25-T1-MPRAGE-1mm brain atlas (fixed image). This registration step was also subject to a visual inspection. Subsequently, both previous transformations (*Φ*_x_ and *Φ*_y_) were used to non-linearly transform all four extracted DTI parameter maps from the patient space into the PD25 atlas space. The affine part of the second transformation (*Φ*_y_ aff) was used to affinely transform the preprocessed T1-weighted scan in the patient space to the PD25 atlas space. Additionally, the displacement fields that resulted from this registration (*Φ*_y_) were used to compute the corresponding log-Jacobian maps using ANTs^66^. Briefly explained, log-Jacobians maps represent how much the volume differs between the atlas and individual preprocessed T1-weighted datasets for each voxel in the atlas space. They are symmetrical around zero with negative values indicating a decrease in volume and positive values indicating an increase in volume with respect to the atlas, which makes them well-suited for training CNNs to capture macro-structural brain information as we have shown in our previous work^14^. The MNI PD25 brain mask was used to remove any undesired volume changes outside of the brain (background voxels) induced by ANTs’ regularizer. To finalize step two, the DTI metrics in the atlas space, the affinely registered T1-weighted scans, and the log-Jacobian maps were all cropped to 160 × 192 × 160 voxels to remove unnecessary background data and reduce the data size for the CNN setup. Lastly, the inverse transformation between the individual T1-weighted scans and the PD25 atlas (Inv *Φ*_y_) was used to warp the cortical and subcortical HO brain regions^38–41^ from the PD25 atlas space to the individual T1-weighted MRI space with the nearest-neighbor interpolation. The HO subcortical atlas includes 21 brain areas, such as the thalamus, hippocampus, and brainstem, and the HO cortical atlas is comprised of 48 brain regions like the subcallosal cortex, frontal cortex, and planum-polare. Each of these regions in subject space was used to compute the corresponding volume and to determine the median MD, FA, RD, and AD values in those regions. To account for different global head sizes, the volumes of the HO brain regions were normalized with the individual’s intracranial volumes (i.e., each structure’s volume/intracranial volume) calculated from every patient’s brain mask created during step two. Median DTI values were selected over mean values to account for potential non-normal distributions and partial volume effects at the border of brain structures. The cortical and subcortical Harvard-Oxford atlas brain regions were only used for the analysis of the saliency maps and for the baseline comparison model using a classical machine learning model but are not required for the training and testing of the deep learning model described below.

### Deep learning and machine learning models

The simple fully convolutional neural (SFCN) network^67^ served as the basis for the deep learning model trained and optimized in this study. This model has obtained state-of-the-art performance in multimodal adult brain age prediction and sex classification using T1-weighted MRI datasets^68,69^. Additionally, the SFCN model has been effectively combined with techniques for computing saliency maps for a variety of applications^14,68,69^. Shortly described, the proposed architecture contained two input layers, one 4D input layer with dimensions of 160 × 192 × 160 × 6 for the input imaging maps (all four 3D DTI parameter maps, the affinely registered 3D T1-weighted scan, the 3D log-Jacobian map) and a second input layer for age and sex; five convolutional blocks, each including a 3D convolutional layer with a 3 × 3 × 3 kernel (same padding), batch normalization, a 2 × 2 × 2 max pooling (same padding), and ReLU activation. A 3D convolutional layer with a 1 × 1 × 1 kernel (same padding), a batch normalization layer, a 3D average pooling layer (same padding), and ReLU activation made up the sixth convolutional block. The individual filter sizes for each convolutional layer were 32, 64, 128, 256, 256, and 64. The seventh and last block contained a dropout layer with a rate of 0.2, the output was flattened and concatenated with age and sex. The result was then passed to an additional dense layer with 16 units and ReLU activation before being transferred to a single classification node with sigmoid activation.

The data were split into 75% training, 10% validation, and 15% testing stratified by age, sex, group (HC and PD), and data origin (study) in each split. This was done to minimize the risk of adding data biases during training and to counteract data imbalances in the individual studies. The proposed model was trained from scratch in an end-to-end fashion and by utilizing all four computed 3D DTI maps (FA, MD, RD, AD), the affinely registered 3D T1-weighted scans, the 3D log-Jacobian map, and age and sex (**CNN**_**COMBINED**_) with randomly initiated weights through 110 epochs following an early stopping condition determined by the model’s performance based on the validation set. The atlas region information was not used in the CNN at any time. The Adam optimizer^70^ was used to optimize a binary cross entropy loss during training. The loss was weighted so that each study received equal weights in terms of data size disparities and patient distribution. These weights were specified as 1 minus the proportion of participants from a particular group (PD or HC) that each study contributed to the total number of datasets in that group (e.g., PPMI gave 222 PD patients, hence the weight was = 1 − (222/611)). In addition, the learning rate was empirically determined to be 0.01, with a beta1 parameter of 0.95, an epsilon parameter of 0.001 for the Adam optimizer, and a batch size of 4. All experiments in this study were implemented on a GeForce RTX 3090 using TensorFlow^71^.

Eight other models, including deep learning and classical machine learning methods, were created for comparison purposes. The first comparison model was trained to investigate how well a CNN model using the 3D DTI maps alone (**CNN**_**DTI**_) but with age and sex as additional features can identify patients with PD. Moreover, we trained six more CNN models, one for each DTI map alone (**CNN**_**FA, MD, RD, and AD**_), one using the preprocessed T1-weighted data only, and one using the log-Jacobian maps (**CNN**_**AFF**_ and **CNN**_**JAC**_). Each of these models also included age and sex as additional features. These six models were trained using the same data splits, architecture, and validation techniques used for the **CNN**_**COMBINED**_. The baseline models were tuned using independent hyperparameters, which can be found in Table 3. Finally, a classical random forest machine learning model^72^ was trained using the extracted tabulated data to enable a comparison with previously developed classical machine learning models^13,23,26,28–30,33,34^. The random forest model was trained using volume and median DTI metric values for each anatomical region in the Harvard-Oxford atlases (**RF**_**COMBINED**_). This model was optimized with the same data splits as the CNN models described above, a batch size of 100, and 200 trees. RELIEFF feature selection with ten nearest neighbors^73^ was used to rank the features based on their importance using the training set to determine the most predictive features, thereby restricting the amount of redundant and non-informative parameters. Iteratively, the least significant feature was removed, the model was re-run, building a new model each time a feature was discarded until only two features remained. RELIEFF was chosen for this task because of its ability to detect non-linear correlations between features and the output class. The model with the best performance within the **RF**_**COMBINED**_ framework was selected to serve as an optimized baseline model representing the combination of traditional feature engineering and machine learning models.

**Table 3:** Hyperparameters for baseline models

Model	Learning rate	Beta 1	Epsilon
CNN_DTI_	1e-5	0.85	0.001
CNN_FA_	0.0003	0.9	1e-7
CNN_MD_	0.0003	0.95	1e-6
CNN_RD_	0.0001	0.9	1e-7
CNN_AD_	0.0003	0.85	1e-6
CNN_JAC_	0.0005	0.85	0.0001
CNN_AFF_	0.003	0.9	0.001

### Saliency map calculation and region of importance identification

Saliency maps were created for the best-performing CNN model (based on the ROC-AUC) to determine which areas of the brain and imaging parameter contributed the most to the classification of HC and PD. These saliency maps were generated using the SmoothGrad method^74^ for each correctly predicted participant from the test set using **CNN**_**COMBINED**_, which performed best overall for the test set (see Results Section). Briefly explained, this method adds random noise from a Gaussian distribution to each test dataset and feeds the noisy images into the trained model. The approach then computes and backpropagates the loss function’s associated partial derivative with respect to the noisy input picture. Consequently, each voxel is allocated one value that indicates its relevance to the model’s output. To smoothen the saliency maps, this method is repeated 25 times with a noise standard deviation of 0.01. The intensities of the saliency maps were then min–max normalized to a range of 0 to 1, and the resulting maps were averaged. To acquire a better understanding of the model’s general behavior, a single population saliency map was constructed for each modality by averaging the subject-specific saliency maps across all correctly classified test subjects. The lower voxel intensity values of the average maps were removed with a 0.5 threshold to focus the analysis on the most important highlighted regions.

The Harvard–Oxford cortical and subcortical atlas brain areas (see Preprocessing Section) were utilized to quantify the computed average saliency maps and determine the most relevant regions for classifying Parkinson’s disease patients across the different MRI modalities. Aside from this post-hoc analysis, the atlas regions were not used in any stage of the model training or testing. The volume proportion of each Harvard-Oxford atlas region covered by the thresholded average saliency maps, as well as the corresponding mean saliency map values were computed and explored for each imaging input map for this purpose. Lastly, micro-structural changes of the brain are known to precede macro-structural changes^13^. Thus, the saliency maps of early patients (PPMI patients in the test set) and non-early patients (rest of the test set) were also computed and analyzed to investigate if DTI metrics are more important for early-onset PD patients.

### Evaluation metrics

The area under the receiver operating characteristic curve was used to evaluate the performance of each trained classifier, which is sensitive to the trade-off between true and false positive rates^21^. Additional metrics including accuracy, specificity, and sensitivity based on a probability threshold (0.5) were also computed as secondary evaluation metrics. Additionally, a paired *t*-test was also conducted to establish statistical significance between the models’ predictions, whereas a *p* value of 0.05 was considered statistically significant.

### Supplementary information

Suplementary Material PDF

Related Manuscript File

## Data Availability

Openly available datasets that support the findings of this study were obtained from the Parkinson’s Progression Markers Initiative (PPMI) database (www.ppmi-info.org/data), the C-BIG repository (https://www.mcgill.ca/neuro/open-science/c-big-repository), and the Alzheimer’s Disease Neuroimaging Initiative (ADNI) database (adni.loni.usc.edu). The ADNI was launched in 2003 as a public–private partnership, led by principal Investigator Michael W. Weiner, MD. The primary goal of ADNI has been to test whether serial magnetic resonance imaging (MRI), positron emission tomography (PET), other biological markers, and clinical and neuropsychological assessment can be combined to measure the progression of mild cognitive impairment (MCI) and early Alzheimer’s disease (AD). For up-to-date information, see www.adni-info.org. The other studies included in this work retain ownership of their scans and are thus not openly available.

## Code availability

The underlying code for this study will be made publicly available to facilitate reproducibility (https://github.com/miltoncamachocamacho/PD-SFCNN).
