# Supplementary material for: Exploiting macro- and micro-structural brain changes for improved Parkinson’s disease classification from MRI data
Source: NPJ Parkinsons Dis. 2024 Feb 26;10:43. doi: 10.1038/s41531-024-00647-9 (PMC10897162; doi:10.1038/s41531-024-00647-9)
Supplement: Supplementary file 1 — Suplementary Material PDF [file 41531_2024_647_MOESM1_ESM.pdf]

Supplementary Table 1 Inclusion/Exclusion criteria of the included studies

Name of Study	Inclusion/Exclusion criteria	
	Parkinson's Disease	Healthy Controls
PPMI	**Detailed inclusion/exclusion criteria in:** https://www.ppmi-info.org/study-design/research-documents-and-sops	**Detailed inclusion/exclusion criteria in:** https://www.ppmi-info.org/study-design/research-documents-and-sops
COMPASS-ND	**Detailed inclusion/exclusion criteria in:** Postuma et al.,[1]	-
PD-MCI Calgary	**Inclusion:**<br>• Non-demented PD patients at stages II or III of Hoehn & Yahr at Time point 1 with or without MCI, men or women, 55 years of age or older.<br>• Willing and able to provide written informed consent.<br>• Willing to provide blood samples, willing to participate in all clinical assessments, willing to have brain MRIs.<br>**Exclusion:**<br>• All participants who met the diagnosis of dementia at Time point 1 as indicated by MMSE of 20 or less and clinical testing. The neuropsychological evaluation always took place before the imaging sessions, in case participants had to be excluded based on their cognitive profile.<br>• All participants taking benzodiazepines were excluded as these can severely impair the performance of cognitive tasks.<br>• Participants with metallic objects in their bodies were not eligible for the study because the strong magnetic field in the scanner could cause these objects to change position and cause injury.<br>• The following criteria was also used as grounds for exclusion, as they have severe impact on cognitive function:<br>○ Alcohol-dependency.<br>○ Presence or history of severe psychiatric disorder, neurological disorder, or stroke.<br>○ General anaesthesia in the past six months.<br>○ History of cerebrovascular disorders.<br>○ More than three concussions or at least 30 min of unconsciousness.	**Inclusion:**<br>• Community volunteers, men, and women, with no history of PD or cognitive or memory complaints, 55 years of age or older.<br>• Willing and able to provide written informed consent.<br>• Willing to provide blood samples, willing to participate in all clinical assessments, willing to have brain MRIs.<br>• Screen negative for MCI.<br>**Exclusion:**<br>Same as for PD
C-Big	**Inclusion:**<br>• People over 18 years old diagnosed with Parkinson's disease at any stage of the disease.	**Inclusion:**<br>• People over 18 years older with no history of PD or a Parkinson Plus Syndrome.<br>• No diagnosis of any neurodegenerative disorder
Hamburg	**Detailed inclusion/exclusion criteria in:** Boelmans et al.,[2]	**Inclusion:**<br>• Healthy controls had no history of central nervous system disease, and their neurological examination was normal. In addition, their MRI brain images were reviewed by 2 experienced blinded neuroradiologists and were reported to be normal
UK Biobank	**Inclusion/exclusion criteria:**<br>• As determined by the ICD code	**Inclusion/exclusion criteria:**<br>• As determined by the ICD code
PD-MCI Montreal	**Detailed inclusion/exclusion criteria in:** Hanganu et al.,[3]	-

ADNI	-	**Detailed inclusion/exclusion criteria in:** https://adni.loni.usc.edu/methods/documents/

Supplementary Table 2 Acquisition information for every included site.

**Name of Study**	**Scanner Manufacturer and Models**	**Magnetic field strength**	**Imaging protocol**
PPMI	Siemens (TrioTim, Verio, Prisma, Biograph_mMR, Espree, Skyra, and Symphony), GE (Signa Hdxt, Signa Excite, Discovery 750, Optima MR 450, Signa Architect, Genesis Signa), and Philips (Achieva, Intera, Achieva_dStream, Ingenia, and Gyroscan NT)	3T – 1.5T	Detailed info in: https://www.ppmi-info.org/study-design/research-documents-and-sops
CCNA	Siemens (TrioTIM, Prima, and Skyra) and GE (Discovery 750)	3T	Detailed info in: https://ccna-biomarkers.readthedocs.io/en/latest/MRI.html
PD-MCI Calgary	GE Discovery 750	3T	Sagittal T1w-3D MPRAGE (TR = 7.2ms, TE = 2.252ms, TI = 600ms, flip angle: 10°, slices 172, field of view: 25.6, acquisition matrix = 256x256, voxel size: 1 x 1 x 1 mm)<br>DWI = 2D spin-echo EPI sequence, 64 diffusion-encoding gradients, b: 1500 s/mm^2, 75 slices, 2mm thickness, in-plane resolution 2x2mm, FoV: 120mm (120x120x77 matrix), TR/TE: 8,000/66 milliseconds, flip angle: 90
C-Big	Siemens Magnetom	3T	Sagittal T1w-3D MPRAGE (TR=2300ms, TE=2.98ms, flip angle=9 degrees, TI=900ms, field of view: 256mm, 192 slices, voxel size: 1 x 1 x 1 mm)<br>DWI = spin-echo EPI sequence, 30 diffusion-encoding gradients, b: 1000 s/mm^2, 70 slices, 2mm thickness, in-plane resolution 2x2mm, FoV: 128mm (128x128x70 matrix), TR/TE: 6,900/64 milliseconds, flip angle: 90
Hamburg	Siemens Skyra	3T	High-resolution T1w-3D MPRAGE (TR=1900ms, TE=2.46ms, flip angle=9 degrees, TI=900ms, image in-plane resolution of 0.94mm^2, and 0.94mm slice thickness)<br>DWI = spin-echo EPI sequence, 20 diffusion-encoding gradients, b: 1000 s/mm^2, 27 slices, 5mm thickness, in-plane resolution 1.875x1.875mm, TR/TE: 4,500/83 milliseconds, flip angle: 90
UK Biobank	Siemens Skyra	3T	Sagittal T1W-3D MPRAGE (TR=2000 ms, TE = 2.01ms, flip angle=8 degrees, 208x256x256 matrix size, voxel size: 1 x 1 x 1 mm)<br>(For more details:<br>https://biobank.ndph.ox.ac.uk/showcase/ukb/docs/brain_mri.pdf<br>https://biobank.ctsu.ox.ac.uk/crystal/ukb/docs/bmri_V4_23092014.pdf)<br>DWI = SE-EPI sequence, 100 diffusion-encoding gradients, b: 1000 s/mm^2; b: 2000 s/mm^2, 72 slices, resolution: 2x2x2mm, FoV: 210mm (104x104x72 matrix), TR/TE: 3,600/92 milliseconds, flip angle: 78
PD-MCI Montreal	Siemens Trio Tim	3T	T1w-3D gradient-echo sequence (TR/TE/TI: 2300/2.91/900 ms, flip angle: 9 degrees, 160 slices, field of view: 256 × 240 mm, matrix: 256 × 240, voxel size: 1 × 1 × 1 mm, 12-channels coil)<br>DWI = 2D spin-echo EPI sequence, 64 diffusion-encoding gradients, b: 700 s/mm^2, 75 slices, 2mm thickness, in-plane resolution 2x2mm, FoV: 128mm (128x128x75 matrix), TR/TE: 12,700/100 milliseconds, flip angle: 90
ADNI	Siemens (TrioTim, Verio, Prisma, Skyra, Biograph_mMR), GE (Discovery MR750, and Signa), Philips (Achieva, Achieva dStream, Ingenia)	3T	Detailed info in: https://adni.loni.usc.edu/methods/mri-tool/mri-analysis/

Supplementary Table 3 Important features used for classical machine learning.

Order of importance	Important features
1	Sex
2	frontal_pole_fa
3	left_cerebral_cortex_fa

4	right_cerebral_cortex_fa
5	lateral_occipital_cortex_superior_division_fa
6	subcallosal_cortex_md
7	subcallosal_cortex_ad
8	subcallosal_cortex_rd
9	age_znorm
10	occipital_pole_fa
11	frontal_medial_cortex_fa
12	postcentral_gyrus_fa
13	precentral_gyrus_fa
14	brain-stem_ad
15	inferior_frontal_gyrus_pars_opercularis_fa
16	inferior_temporal_gyrus_anterior_division_fa
17	right_cerebral_cortex
18	superior_frontal_gyrus_fa
19	middle_temporal_gyrus_temporooccipital_part_fa
20	temporal_pole_fa
21	lateral_occipital_cortex_inferior_division_fa
22	middle_temporal_gyrus_posterior_division
23	occipital_pole_ad
24	occipital_pole_md
25	lingual_gyrus_fa
26	left_cerebral_white_matter_ad
27	temporal_fusiform_cortex_anterior_division_fa
28	brain-stem_md
29	middle_frontal_gyrus_fa
30	occipital_pole_rd
31	superior_parietal_lobule_fa
32	inferior_temporal_gyrus_temporooccipital_part_fa
33	left_cerebral_cortex
34	inferior_frontal_gyrus_pars_opercularis
35	temporal_occipital_fusiform_cortex
36	frontal_medial_cortex_rd
37	brain-stem_rd
38	icv
39	angular_gyrus_fa
40	left_pallidum_ad
41	right_amygdala_ad
42	frontal_medial_cortex_md
43	heschls_gyrus_fa
44	supramarginal_gyrus_posterior_division_fa
45	right_caudate_fa
46	right_pallidum_fa
47	left_cerebral_white_matter_fa
48	temporal_occipital_fusiform_cortex_fa
49	right_putamen_fa
50	frontal_operculum_cortex
51	left_caudate_fa
52	parahippocampal_gyrus_anterior_division_fa
53	right_cerebral_white_matter
54	right_cerebral_white_matter_ad
55	supramarginal_gyrus_anterior_division_fa
56	left_pallidum_fa
57	right_amygdala_md
58	central_opercular_cortex
59	right_cerebral_white_matter_fa
60	precuneous_cortex_fa
61	left_thalamus_ad

62	left_cerebral_white_matter_md
63	temporal_fusiform_cortex_anterior_division
64	frontal_medial_cortex_ad
65	right_pallidum
66	cuneal_cortex_fa
67	left_cerebral_white_matter_rd
68	planum_temporale_fa
69	frontal_orbital_cortex_md
70	insular_cortex_fa
71	inferior_frontal_gyrus_pars_triangularis_fa
72	left_pallidum_md
73	left_thalamus_md
74	paracingulate_gyrus_fa
75	cingulate_gyrus_anterior_division_fa
76	frontal_orbital_cortex_rd
77	inferior_temporal_gyrus_posterior_division_fa
78	frontal_orbital_cortex_fa
79	middle_temporal_gyrus_anterior_division
80	right_thalamus_ad
81	frontal_orbital_cortex_ad
82	left_thalamus_rd
83	left_thalamus_fa
84	precentral_gyrus
85	precentral_gyrus_ad
86	parahippocampal_gyrus_anterior_division_ad
87	parahippocampal_gyrus_anterior_division_md
88	parahippocampal_gyrus_anterior_division_rd
89	parahippocampal_gyrus_posterior_division_rd
90	superior_temporal_gyrus_anterior_division_fa
91	left_pallidum_rd
92	planum_polare
93	right_thalamus_md
94	precentral_gyrus_md
95	precentral_gyrus_rd
96	left_amygdala_ad
97	lateral_occipital_cortex_inferior_division_ad
98	supracalcarine_cortex
99	left_amygdala_md
100	parietal_operculum_cortex_fa
101	temporal_fusiform_cortex_posterior_division_fa
102	superior_temporal_gyrus_posterior_division
103	brain-stem
104	postcentral_gyrus_rd
105	right_thalamus_fa
106	supramarginal_gyrus_anterior_division
107	cuneal_cortex_ad
108	postcentral_gyrus_md
109	right_amygdala_rd
110	right_accumbens_fa
111	parahippocampal_gyrus_posterior_division_md
112	right_cerebral_white_matter_rd
113	frontal_pole_ad
114	right_cerebral_white_matter_md
115	left_pallidum
116	insular_cortex_ad
117	right_thalamus_rd
118	left_putamen_fa
119	brain-stem_fa

120	postcentral_gyrus_ad
121	postcentral_gyrus
122	intracalcarine_cortex_fa
123	cuneal_cortex
124	paracingulate_gyrus_ad
125	cingulate_gyrus_posterior_division_fa
126	inferior_temporal_gyrus_temporooccipital_part
127	middle_temporal_gyrus_temporooccipital_part_ad
128	right_pallidum_ad
129	occipital_fusiform_gyrus_fa
130	inferior_temporal_gyrus_anterior_division_rd
131	frontal_operculum_cortex_fa
132	frontal_pole
133	middle_temporal_gyrus_posterior_division_rd
134	subcallosal_cortex_fa
135	paracingulate_gyrus_md
136	left_cerebral_cortex_ad
137	superior_temporal_gyrus_posterior_division_fa
138	paracingulate_gyrus_rd
139	juxtapositional_lobule_cortex_fa
140	superior_frontal_gyrus_rd
141	middle_temporal_gyrus_posterior_division_ad
142	middle_temporal_gyrus_posterior_division_fa
143	parahippocampal_gyrus_posterior_division_fa
144	middle_temporal_gyrus_posterior_division_md
145	left_cerebral_white_matter
146	planum_polare_fa
147	right_cerebral_cortex_ad
148	left_amygdala_rd
149	superior_frontal_gyrus_md
150	supracalcarine_cortex_fa
151	inferior_temporal_gyrus_anterior_division_md
152	lateral_occipital_cortex_superior_division_ad
153	temporal_fusiform_cortex_posterior_division_ad
154	middle_frontal_gyrus_ad
155	inferior_temporal_gyrus_posterior_division_ad
156	temporal_pole_rd
157	central_opercular_cortex_fa
158	frontal_pole_rd
159	lingual_gyrus

References

1. Postuma RB, Berg D, Stern M, et al. MDS clinical diagnostic criteria for Parkinson's disease. *Movement Disorders*. 2015;30(12):1591-1601. doi:10.1002/MDS.26424

2. Boelmans K, Holst B, Hackius M, et al. Brain iron deposition fingerprints in Parkinson's disease and progressive supranuclear palsy. *Movement Disorders*. 2012;27(3):421-427. doi:10.1002/MDS.24926

3. Hanganu A, Bedetti C, Degroot C, et al. Mild cognitive impairment is linked with faster rate of cortical thinning in patients with Parkinson's disease longitudinally. *Brain*. 2014;137(4):1120-1129. doi:10.1093/BRAIN/AWU036
