# Supplementary material for: Exploiting macro- and micro-structural brain changes for improved Parkinson’s disease classification from MRI data
Source: NPJ Parkinsons Dis. 2024 Feb 26;10:43. doi: 10.1038/s41531-024-00647-9 (PMC10897162; doi:10.1038/s41531-024-00647-9)
Supplement: Supplementary file 2 — Related Manuscript File [file 41531_2024_647_MOESM2_ESM.pdf]

Corresponding author(s): Milton Camacho
Last updated by author(s): Oct 23, 2023

Reporting Summary

Nature Portfolio wishes to improve the reproducibility of the work that we publish. This form provides structure for consistency and transparency in reporting. For further information on Nature Portfolio policies, see our Editorial Policies and the Editorial Policy Checklist.

Statistics

For all statistical analyses, confirm that the following items are present in the figure legend, table legend, main text, or Methods section.

n/a	Confirmed	
☐	☒	The exact sample size (*n*) for each experimental group/condition, given as a discrete number and unit of measurement
☒	☐	A statement on whether measurements were taken from distinct samples or whether the same sample was measured repeatedly
☐	☒	The statistical test(s) used AND whether they are one- or two-sided<br>*Only common tests should be described solely by name; describe more complex techniques in the Methods section.*
☒	☐	A description of all covariates tested
☒	☐	A description of any assumptions or corrections, such as tests of normality and adjustment for multiple comparisons
☒	☐	A full description of the statistical parameters including central tendency (e.g. means) or other basic estimates (e.g. regression coefficient) AND variation (e.g. standard deviation) or associated estimates of uncertainty (e.g. confidence intervals)
☒	☐	For null hypothesis testing, the test statistic (e.g. *F*, *t*, *r*) with confidence intervals, effect sizes, degrees of freedom and *P* value noted<br>*Give P values as exact values whenever suitable.*
☒	☐	For Bayesian analysis, information on the choice of priors and Markov chain Monte Carlo settings
☒	☐	For hierarchical and complex designs, identification of the appropriate level for tests and full reporting of outcomes
☒	☐	Estimates of effect sizes (e.g. Cohen's *d*, Pearson's *r*), indicating how they were calculated

Our web collection on statistics for biologists contains articles on many of the points above.

Software and code

Policy information about availability of computer code

Data collection	Retrospective data analysis, therefore, no software was used.
Data analysis	Advanced Normalization Tools v2.4.0, Python 3.9, Tensorflow 2.11, Tractoflow 2.4.0

For manuscripts utilizing custom algorithms or software that are central to the research but not yet described in published literature, software must be made available to editors and reviewers. We strongly encourage code deposition in a community repository (e.g. GitHub). See the Nature Portfolio guidelines for submitting code & software for further information.

Data

Policy information about availability of data

All manuscripts must include a data availability statement. This statement should provide the following information, where applicable:

- Accession codes, unique identifiers, or web links for publicly available datasets
- A description of any restrictions on data availability
- For clinical datasets or third party data, please ensure that the statement adheres to our policy

Openly available datasets that support the findings of this study include PPMI through ppmi-info.org, ADNI through www.adni-info.org, and C-BIG (https://www.mcgill.ca/neuro/open-science/c-big-repository). The other studies included in this work retain ownership of their scans and are thus not openly available.

Research involving human participants, their data, or biological material

Policy information about studies with human participants or human data. See also policy information about sex, gender (identity/presentation), and sexual orientation and race, ethnicity and racism.

Reporting on sex and gender	No sex or gender analysis were reported in order to avoid extending our study.
Reporting on race, ethnicity, or other socially relevant groupings	No race, ethnicity, or social relevant grouping analysis was made, this was due to the lack of this information for the mayority of the participants included.
Population characteristics	Only age and sex were included due to the lack of other characteristics available.
Recruitment	No recruitment was performed due to the retrospective nature of the study.
Ethics oversight	Conjoint Health Research Ethics Board at the University of Calgary.

Note that full information on the approval of the study protocol must also be provided in the manuscript.

Field-specific reporting

Please select the one below that is the best fit for your research. If you are not sure, read the appropriate sections before making your selection.

☒ Life sciences ☐ Behavioural & social sciences ☐ Ecological, evolutionary & environmental sciences

For a reference copy of the document with all sections, see nature.com/documents/nr-reporting-summary-flat.pdf

Life sciences study design

All studies must disclose on these points even when the disclosure is negative.

Sample size	The largest balanced sample size was sought given that large data is required to train deep learning models.
Data exclusions	Inclusion and exclusion criteria were described in the supplementary material.
Replication	Seeds were used to increase the reproducibility off our trained deep learning networks with good reproducibility.
Randomization	Randomization followed a stratified split based on study, group, age, and sex.
Blinding	Blinding was not relevant for our study since we were working with a retrospectively collected dataset and deep learning.

Reporting for specific materials, systems and methods

We require information from authors about some types of materials, experimental systems and methods used in many studies. Here, indicate whether each material, system or method listed is relevant to your study. If you are not sure if a list item applies to your research, read the appropriate section before selecting a response.

Materials & experimental systems

n/a	Involved in the study	
☒	☐	Antibodies
☒	☐	Eukaryotic cell lines
☒	☐	Palaeontology and archaeology
☒	☐	Animals and other organisms
☒	☐	Clinical data
☒	☐	Dual use research of concern
☒	☐	Plants

Methods

n/a	Involved in the study	
☒	☐	ChIP-seq
☒	☐	Flow cytometry
☐	☒	MRI-based neuroimaging

Plants

Seed stocks: n/a

Novel plant genotypes: n/a

Authentication: n/a

Magnetic resonance imaging

Experimental design

Design type: n/a

Design specifications: n/a

Behavioral performance measures: n/a

Acquisition

Imaging type(s): Structural

Field strength: 3 Tesla

Sequence & imaging parameters: Multi-center study: these were defined in great detail in our supplementary material.

Area of acquisition: Whole brain

Diffusion MRI: ☒ Used ☐ Not used

Parameters: Multi-center study: these were defined in great detail in our supplementary material.

Preprocessing

Preprocessing software: HD-BET v1, Advanced Normalization Tools v2.4.0, Tractoflow 2.4.0 for DTI, Convert 3D

Normalization: Non-linear registration using rigid, affine, and non-linear components to transform Nifty images into the template's space.

Normalization template: MNI PD25 Atlas.

Noise and artifact removal: Skull stripping, non-local denoising, eddy current correction, and N4 bias correction

Volume censoring: The spatial normalization through image registration served as volume censoring.

Statistical modeling & inference

Model type and settings: 3D Convolutional neural network as a predictive model

Effect(s) tested: n/a

Specify type of analysis: ☐ Whole brain ☐ ROI-based ☒ Both

Anatomical location(s): Anatomical locations where defined based on the HarvardOxford cortical and subcortical atlases.

Statistic type for inference (See Eklund et al. 2016): n/a

Correction: n/a

Models & analysis

n/a | Involved in the study

- [x] n/a — [ ] Functional and/or effective connectivity
- [x] n/a — [ ] Graph analysis
- [ ] n/a — [x] Multivariate modeling or predictive analysis

Multivariate modeling and predictive analysis

Independent variables were the different three-dimensional input maps which where are used to train our deep learning model. Feature extraction and dimensionality reduction were automatically performed by the convolutional neural network. We performed a stratified train/validation/and testing split evaluated by area under the curve and additional metrics like accuracy, specificity, and sensitivity.
